# What we can learn from animal models about cerebral multi-morbidity

**DOI:** 10.1186/s13195-015-0097-2

**Published:** 2015-01-29

**Authors:** Siân Baker, Jürgen Götz

**Affiliations:** Clem Jones Centre for Aging Dementia Research, Queensland Brain Institute, The University of Queensland, Upland Road, Building 79, St Lucia Campus, Brisbane, QLD 4072 Australia

## Abstract

Late-onset diseases such as Alzheimer’s disease, Parkinson’s disease, or frontotemporal lobar degeneration are considered to be protein-folding disorders, with the accumulation of protein deposits causing a gain-of-toxic function. Alzheimer’s disease is characterized by two histological hallmark lesions: amyloid-β-containing plaques and tau-containing neurofibrillary tangles. However, signature proteins, including α-synuclein, which are found in an aggregated fibrillar form in the Lewy bodies of Parkinson’s disease brains, are also frequently found in Alzheimer’s disease. This highlights the fact that, although specific aggregates form the basis for diagnosis, there is a high prevalence of clinical overlap between neuropathological lesions linked to different diseases, a finding known as cerebral co- or multi-morbidity. Furthermore, the proteins forming these lesions interact, and this interaction accelerates an ongoing degenerative process. Here, we review the contribution that transgenic animal models have made to a better mechanistic understanding of the causes and consequences of co- or multi-morbidity. We discuss selected vertebrate and invertebrate models as well as the insight gained from non-transgenic senescence-accelerated mouse-prone mice. This article is part of a series on ‘Cerebral multi-morbidity of the aging brain’.

## Introduction

A unifying feature of the pathology of neurodegenerative diseases is the accumulation of misfolded proteins that form insoluble aggregates in both the intra- and extra-cellular space of the central nervous system. Traditionally, the pathological classification of neurodegenerative diseases has been based on the principal proteins that are present in these aggregates and their localization to distinct brain areas. However, it is rare for the deposited proteins to be unique to one disease. In reality, although specific aggregates form the basis for diagnosis, there is a high prevalence of clinical overlap between neuropathological lesions linked to different diseases, a finding known as cerebral co- or multi-morbidity [1]. Clinical and neuropathological findings are discussed in detail in the articles that accompany our review as part of a series on ‘Cerebral multi-morbidity of the aging brain’. Here, we focus on the insight provided by animal models.

Of all the dementias, Alzheimer’s disease (AD) is the most prevalent, accounting for approximately two thirds of all cases. Neuropathologically, in addition to neuron and synapse loss, the disease is characterized by the presence of amyloid-β (Aβ)-containing plaques—with Aβ being proteolytically derived from the larger amyloid precursor protein (APP)—as well as tau-containing neurofibrillary tangles (NFTs) and neuropil threads. It is, however, becoming increasingly clear that these lesions often co-exist with other forms of protein aggregates. In fact, about two thirds of aged human AD brains contain additional non-AD pathologies [2-6]. These include protein aggregates of α-synuclein, a defining feature of Parkinson’s disease (PD) and dementia with Lewy bodies (DLB), as well as transactive response DNA-binding protein 43 kDa (TDP-43) that forms aggregates in subtypes of frontotemporal lobar degeneration (FTLD) and amyotrophic lateral sclerosis (ALS), the latter also known as motor neuron disease [7,8].

Age is the most important risk factor for AD. On the one hand, the hypothesis has been formulated that AD is an inevitable manifestation of senescence in that the disease, with its neuropathological signatures, is considered a normal phenomenon of aging [9]. On the other hand, the pattern of neuronal loss was shown to differ between normal aging and AD, suggesting that the latter is not an inevitable consequence of the former [10]. Interestingly, however, a recent study indicates that much of late-life cognitive decline (60%) is not due to common neurodegenerative pathologies such as plaques and NFTs, suggesting that other important determinants are still to be identified [11].

In animals, age-related cognitive impairment or even an AD-like pathology is seen in species that reach an advanced age. In fact, all non-human primate species examined to date have been shown to display NFTs, or Aβ plaques, or both [12,13] (and references therein). Further studies in these species are warranted. Plaques have been reported in the brains of cetaceans (such as whales), birds, fish, carnivorans (such as bears), and ungulates, and NFTs have been reported in the latter two groups [14].

## Modelling plaques and neurofibrillary tangles in animals

Unfortunately, most of the above species are not easily amenable to experimental manipulation, and the animal species traditionally used in laboratory settings, such as mice, flies, or worms, do not naturally develop the protein aggregates seen in AD, in part because of their relatively short life span [14]. Nonetheless, these species have been successfully developed into experimental animal models for AD by expressing pathogenic mutations that are found in the genes encoding APP, presenilin-1, and presenilin-2 in familial early-onset AD as well as by expressing pathogenic mutations in the tau-encoding *MAPT* (microtubule-associated protein tau) gene found in familial cases of FTLD (FTDP-17t) [15].

It took several attempts for the research community to succeed in reproducing the end-stage lesions of AD, NFTs and plaques in transgenic mouse models [15]. The discovery of pathogenic mutations, such as those in the *APP* and *MAPT* genes, together with the use of stronger promoters and inducible systems, made possible the reproduction of plaques and NFTs at reasonable numbers and at a reasonable age. Overexpression of wild-type forms of human tau did not reproduce NFTs [16-19] unless the mice reached a high age [20]. Similarly, crossing wild-type human tau transgenic mice with mice carrying the Osaka mutation in APP (E693Δ) resulted in NFT formation at only 18 months of age [21]. However, expression of FTDP-17t mutant forms of tau resulted in a much earlier onset of NFT formation [22,23], and by using an inducible system for transgene expression, the initiation of massive NFT formation has been achieved as early as 2.5 months of age [24].

Small-animal models such as the fruit fly *Drosophila melanogaster* and the roundworm *Caenorhabditis elegans* are useful tools for investigating human disorders, as the genes implicated in human disease have homologues in the invertebrates and because many signalling pathways are conserved. For a review of the currently available models of neurodegeneration in *C. elegans*, see [25]. An advantage of *C. elegans* lies in the fact that the biological function of, for example, the tau homologue Ptl-1 can be studied without the complication of functional redundancy that is observed in mammals (where tau, MAP2, and MAP4 have partly overlapping functions) [26]. In *Drosophila*, the expression of human wild-type and mutant forms of proteins with a role in AD, PD, and FTLD has advanced our understanding of the role of these proteins under physiological and pathological conditions. Examples of such models are α-synuclein or tau transgenic flies [27,28].

Interestingly, work in the roundworm *C. elegans* has shown that the expression of both normal and FTDP-17t mutant tau results in neurodegeneration and defective neurotransmission but that the pathology is more severe in the latter [29]. One of the key advantages of the *C. elegans* system is the possibility of rapid and comparably cheap modifier screens; this has led, for example, to the identification of *sut-2* as being required for tau neurotoxicity [30]. Earlier work in *Drosophila* had shown that tau-dependent neurodegeneration can occur in the absence of NFT formation [28] and that neuronal expression of wild-type tau in the absence of mutations can cause learning and memory deficits [31]. Several studies have addressed the individual roles of distinct phosphorylation sites of tau, rather than hyperphosphorylation *per se*, but more studies are required to pinpoint the role of specific tau phosphorylation events and tau isoforms in disease [32,33]. Importantly, highly phosphorylated tau firstly exhibits significantly reduced binding to microtubules and secondly participates in a pathogenic interaction with normal tau, sequestering it away from microtubules [34].

Studies in invertebrates and vertebrates have collectively identified several modes of tau and Aβ dysfunction and how this results in neurodegeneration [35,36]. Despite the insight into pathomechanisms provided by these models, transgenic approaches have met with criticism because of (i) unphysiologically high protein levels that are caused, for example, by the integration of multiple transgene copies into the genome, (ii) an altered brain area specificity and subcellular expression pattern of the transgene compared with the endogenous gene because of the use of an exogenous promoter, and (iii) disruption or alteration of endogenous gene expression because of insertion of the transgene into the host genome. Consequently, several groups have pursued alternative strategies. For example, more recently, a knock-in approach was used to introduce the P301L mutation of tau into the murine *MAPT* locus. Although these mice failed to develop a mature tau pathology [37], this does not preclude their use in dissecting early pathomechanisms, and it is possible, with the advent of new gene-editing methods, that these models can be further refined [38]. Additional approaches have exploited mice, such as the senescence-accelerated SAMP (senescence-accelerated mouse-prone) strain, that are characterized by accelerated aging [39]. Together with a series of related senescence-accelerated mice, the SAMP strains were established 40 years ago by conventional inbreeding of AKR/J-derived mice that displayed features of accelerated aging such as hair loss, reduced activity, shortened life expectancy, lordokyphosis (increased curvature of the spine), and periophthalmic problems [40]. Littermates of mice that did not show a senescence-associated phenotype were also inbred to generate senescence-resistant, longer-lived senescence-accelerated mouse resistant (SAMR) mice, of which the SAMR1 strain is commercially available. SAMP strains exhibit an early onset of age-related decline in their immune system such as thymic involution, loss of CD4^+^ T cells, impaired helper T-cell function, decreased antibody-forming capacity, dysfunction of antigen-presenting cells, decreased natural killer activity, increased auto-antibodies, and increased susceptibility to viral infection [41].

SAMP8 is the strain that has been most extensively analyzed in relation to cognitive functions [42]. These mice are neuropathologically characterized by oxidative changes similar to those found in the AD brain [43]. Furthermore, they have an impaired glucose metabolism [44] and exhibit age-dependent reductions in various receptors, including the NMDA receptor [45]. Tau was found to be pathologically phosphorylated in this strain, but filament formation and NFT formation have not been reported, indicating that the SAMP8 mice present with an early tau pathology [46]. Staining with Aβ-specific antibodies suggested Aβ deposition in these mice [47,48]; however, unlike the human sequence for the Aβ precursor protein APP, the murine protein lacks the amino acids that are required to generate Aβ, and as a result these deposits have been termed ‘Aβ-like’ [47]. A more recent study reported the presence of Aβ granules in the hippocampus, which contained also tau but not α-synuclein [49]. Another study demonstrated elevated α-synuclein levels in SAMP8 mice compared with senescence-resistant SAMR1 mice, but aggregation of neither tau nor α-synuclein was reported in these animals [50]. Together, these findings limit the use of non-transgenic models such as the SAMP mice to study multi-morbidity, in part because authentic end-stage lesions do not form.

## Comorbidity addressed in animal models

**Figure 1:**
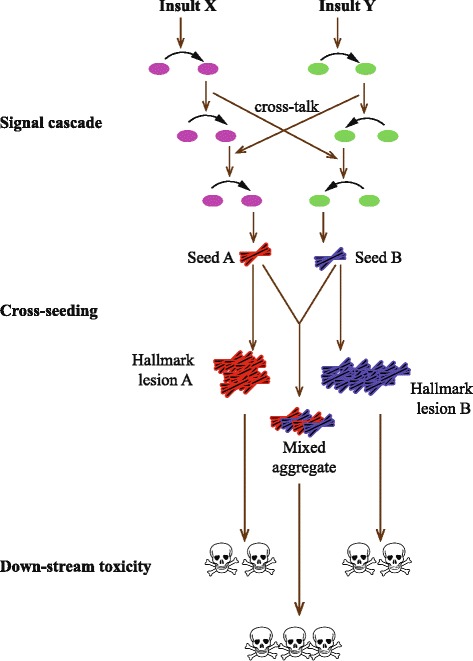
**Potential modes of comorbidity.** In neurodegenerative disorders there are protein insults considered imperative to the development and characterization of a specific disease (hallmark lesion A) and additional protein pathology that is traditionally classified to another disease state (hallmark lesion B). There are several potential mechanisms by which cross-talk may be occurring between these separate lesions to result in comorbidity: i) at the level of the initial pathogenic signalling cascades which results in the formation of seeds A and B, respectively, (ii) through the ability of one pathological protein (which could range from a post-translational modification state through to a mature aggregate) to induce pathological changes in the state of a protein implicated in another disease, which may also lead to the formation of mixed aggregates, and (iii) a convergence onto mutual cell death pathways.

AD and PD are associated with the cerebral accumulation of Aβ/tau and α-synuclein, respectively. Because many patients have clinical and pathological features of both diseases, this raises several possibilities: (i) the pathogenetic pathways overlap; (ii) the presence of one pathologically altered protein (be it in its post-translational modified form or be it present as oligomer, fibril, or microscopically visible aggregate) causes pathological changes to a protein implicated in the other disease, which also includes the formation of mixed aggregates; and finally (iii) synergistic effects exist of Aβ and α-synuclein with regard to downstream toxicity (Figure 1).

Several *in vitro* studies have shown that these particular proteins cross-talk and cause each other’s aggregation. For example, Aβ and α-synuclein can form dimers that dock on the plasma membrane and then incorporate additional α-synuclein molecules, leading to the formation of more stable pentamers and hexamers that adopt a ring-like structure, causing increased calcium influx [51]. These interactions can result in oxidative stress, lysosomal leakage, and mitochondrial dysfunction, as has been discussed in detail by Crews and colleagues [52]. These authors suggested that hybrid molecules of Aβ and α-synuclein might embed not only in the plasma membrane but also in membranes of mitochondria and lysosomes, where they would form nanopore-like structures, which result in abnormal ion conductance. A recent study with implications for DLB showed that the metabotropic glutamate receptor mGluR5 has a role in mediating Aβ oligomer toxicity in hippocampal neurons and that the accumulation of α-synuclein fragments makes these cells more vulnerable [53].

We will now discuss how α-synuclein interacts with tau/Aβ *in vivo* and how this knowledge informs our understanding of comorbidity in AD. In 1993, α-synuclein was identified as the non-Aβ component of AD amyloid [54], and subsequently a plaque-associated α-synuclein pathology was reported in aged amyloid-depositing mutant APP transgenic Tg2576 mice [55]. Although there was limited tau pathology, the mice displayed frequent neurites that were both ubiquitin- and α-synuclein-positive. When mutant APP transgenic mice were crossed with α-synuclein overexpressing mice, α-synuclein oligomer formation was significantly increased in the double-transgenic animals [56]. The mice had severe deficits in learning and memory, developed motor deficits earlier than α-synuclein single-transgenic mice, and showed prominent age-dependent degeneration of cholinergic neurons and presynaptic terminals. They also had more α-synuclein-immunoreactive neuronal inclusions than observed in α-synuclein single-transgenic mice. In addition, some of these inclusions were fibrillar in nature, whereas all inclusions in the single-transgenic mice were amorphous. To address not only whether Aβ has an impact on α-synuclein pathology but also whether the inverse could be true, the Tg2576 strain was crossed onto an α-synuclein knockout background. This resulted in an increased amyloid plaque load, leading the authors to speculate that α-synuclein is not involved in the seeding of the plaques but rather that it suppresses the progression of plaque pathology at advanced stages of the disease [57].

The impact that α-synuclein expression has on tau has also been addressed in transgenic mouse models. Giasson and colleagues [58] showed that co-incubation of tau and α-synuclein promotes the fibrillization of both proteins. Using negative contrast electron microscopy, they further demonstrated the presence of bundled filaments that were labelled with antibodies for both proteins. They also investigated the formation of tau inclusions in A53T mutant α-synuclein transgenic mice and found that, compared with single-transgenic mice, aggregate formation in α-synuclein/P301L tau bigenic mice was accelerated by 6 months. Increased hyperphosphorylation of tau was observed in several additional α-synuclein transgenic mouse models [59-61], and a study in E46K mutant α-synuclein transgenic mice revealed that two pathological phospho-epitopes of tau, AT100 and PHF1 (which is a target of several tau immunization studies), were induced by α-synuclein [61]. The abundance of tau inclusions in the E46K transgenic mice was greater than observed previously in A53T human α-synuclein transgenic mice [58]. One of the kinases implicated in α-synuclein-mediated tau hyperphosphorylation is glycogen synthase kinase 3β (GSK-3β), one of the major tau kinases. It was shown by co-immunoprecipitation that α-synuclein, GSK-3β, and tau phosphorylated at the PHF1 epitope pSer396/404 exist as a heterotrimeric complex in human SH-SY5Y neuroblastoma cells [62]. The crosstalk of α-synuclein and tau was further addressed in *Drosophila*, where misexpression of wild-type α-synuclein was found to enhance a tau-mediated rough eye phenotype and apoptotic cell death in the eye. Similarly, α-synuclein increased tau-dependent abnormal microtubule organization and axonal transport impairment, together with an enhanced tau-induced motor phenotype [63]. Interestingly, the study also found that co-expression of α-synuclein and tau led to decreases in synapsin (a synaptic vesicle-associated phosphoprotein) in synaptic boutons, resulting in synaptic apposition defects consistent with synaptic retraction.

Another study addressed the mechanism by which human tau (a strong risk factor for PD) predisposes an individual to PD [64]. This study found that expression of human wild-type tau was sufficient to disrupt the survival of dopaminergic neurons in a *Drosophila* model and to cause a progressive impairment of motor and learning behaviours. Interestingly, it also demonstrated that, contrary to the common notion that hyperphosphorylated tau aggravates toxicity, the degeneration of dopaminergic neurons was alleviated by expressing a pseudo-hyperphosphorylated form of tau, E14. Several studies used *Drosophila* to better understand the role of APP/Aβ in neurodegeneration [65,66] and more specifically to demonstrate that Aβ exacerbates tau pathology [67].

After the finding that Aβ toxicity in AD can be dramatically reduced by removing tau [68,69], whether this holds true for α-synuclein was also addressed. However, in two PD models—one pharmacological (by striatal injection of 6-hydroxydopamine) and the other a human wild-type α-synuclein transgenic strain—tau reduction did not prevent the motor deficits that characterize these models [70].

To determine how the three key players in AD/PD—Aβ, tau, and α-synuclein—interact, the A53T mutant α-synuclein transgene was introduced into 3xTg-AD mice, a strain characterized by both plaque and NFT pathology [71]. As in human disease, the mice developed both DLB and AD pathologies. Lewy body-like pathology was increased upon co-expression of APP and tau. Tau solubility was decreased and its phosphorylation increased in the crossbred mice, as were levels of detergent-insoluble Aβ (observed for both the Aβ_40_ and Aβ_42_ species). Moreover co-expression of the three proteins accelerated the cognitive decline, with evidence that α-synuclein exacerbated cognitive deficits not only in the acquisition of spatial recognition memory but also in the retention of memory. It was further found that accumulation of α-synuclein alone could significantly disrupt cognition. A different result was reported in a crossbreed of three strains, A53T α-synuclein mutant mice, Tg2576 and a P264L presenilin-1 knock-in strain that further promotes Aβ plaque formation. Here, despite the accumulation of dystrophic neurites that were positive for hyperphosphorylated α-synuclein both within and surrounding Aβ plaques, no additional α-synuclein pathologies were observed. It was concluded that Aβ deposits can cause the local aggregation of α-synuclein but that this does not lead to a more extensive α-synuclein pathology [72].

Considering evidence that soluble, non-fibrillar Aβ (and tau) may be the more neurotoxic species, Larson and colleagues [73] assessed the putative role of soluble α-synuclein in AD. They first showed that there is an approximately twofold increase in monomeric, intracellular α-synuclein in brains from AD patients compared with normal controls and subjects suffering from mild cognitive impairment. This accumulation was found to be independent of Lewy body formation. Interestingly, mRNA levels were also increased approximately twofold in AD patients compared with controls, suggesting the involvement of imbalanced synuclein gene expression. The level of soluble α-synuclein was linked to AD-associated cognitive impairment and was also a good predictor of AD-related impairment. When transgenic mice were analyzed, neither of the two APP mutant lines, Tg2576 and J20, aged between 1 and 17 months, presented with detectable changes in soluble α-synuclein. To test whether expression of human tau is required for the regulation of α-synuclein expression, soluble α-synuclein protein levels were compared in Tg2576 mice, P301L tau over-expressing rTg4510 mice, and Tg2576 × rTg4510 mice. This revealed an approximately twofold increase in soluble α-synuclein at 8 months in Tg2576 × rTg4510 mice, whereas no obvious changes were found in rTg4510 mice across all age groups. These findings indicate that a synergism between Aβ/APP and human tau is required to upregulate α-synuclein expression levels.

An exciting study published in 2013 revealed distinct α-synuclein strains that differentially promote tau inclusions in neurons [74]. Based on the use of exogenous pre-formed fibrils (termed ‘pffs’) of α-synuclein, two strains (A and B) were generated with a differential ability to cross-seed tau aggregation in cultured neurons. Furthermore, stereotaxic injections of the hippocampus of P301S mutant tau transgenic PS19 mice revealed that differential cross-seeding occurs *in vivo*. At 3 months post-injection, only rare cells showed abnormal accumulation of hyperphosphorylated tau, recognized by the AT8 antibody near the injection site of strain A-inoculated mice, whereas numerous neurons bearing AT8-positive tau inclusions were observed in strain B-injected mice around the same area. Moreover, strain B-injected mice not only displayed significantly more tau inclusions throughout the hippocampus, including regions that were more rostral and caudal to the injection site, but also consistently showed phospho-tau aggregates in the contralateral hippocampus and even the locus coeruleus, a brainstem structure distant from the injection site, indicating the presence of transmission of tau pathology cross-seeded by α-synuclein pffs. The differential induction of tau aggregates was further confirmed with a set of antibodies to detect pathological conformations of tau [74].

To date, only a few studies have addressed the effect that other proteins implicated in neurodegeneration have on Aβ, tau, and α-synuclein. Cross-rescue experiments and co-expression models using TDP-43 and FUS (fused in sarcoma) transgenic flies have provided evidence for a genetic interaction of the two proteins in a common pathway, suggesting a convergence of molecular pathways influencing FTLD (and ALS) pathology [75,76]. It has also been shown that inoculation of the brains of α-synuclein transgenic mice with prions (PrP^Sc^) exacerbates the α-synuclein pathology. Remarkably, prion pathology was unmodified by the presence of α-synuclein [77]. Of the proteins other than tau that form aggregates in FTLD, TDP-43, and FUS, only TDP-43 has been analyzed in transgenic mouse models of tauopathy [78]. Cytoplasmic accumulation of phosphorylated TDP-43 was specifically found in two tau transgenic models (P301L 0N4R-expressing rTg4510 mice and JNPL3 mice), but TDP-43 pathology was absent in mouse models of Aβ deposition (TgCRND8, Tg2576, and Tg2576 x P264L PS1 knock-in), α-synucleinopathy (A53T-expressing M83 mice and E46K-expressing M47 mice), or Huntington’s disease (N586-82Q-C63 model). These data demonstrate that the neurodegenerative cascade associated with a primary tauopathy in tau transgenic mice can also promote TDP-43 abnormalities.

## Conclusions

What are possible explanations for co- or multi-morbidity and what have animal models contributed to a better understanding of this? As it stands, late-onset diseases are mainly protein-folding diseases, with the accumulation of protein deposits causing a gain-of-function proteotoxicity [79]. The concept that has been put forward is that the proteostasis machinery is overwhelmed when there is a chronic elevation of misfolded proteins. Molecular chaperones and other components of the ‘clearance machinery’ become trapped in the aggregates and this compromises the re-folding of other aggregation-prone proteins and facilitates their aggregation [79]. Alternatively, filamentous aggregates that are composed of one protein may directly cross-seed other amyloidogenic proteins because of potentially shared structural features of amyloid fibrils [80]. As has been argued for the interaction of α-synuclein and tau, α-synuclein might alter the conformation or solubility of tau in brains with tau inclusions, even in the absence of an obvious α-synuclein pathology. As only minute amounts of amyloidogenic α-synuclein seeds may be required, it is possible that they are undetectable with current methods or, alternatively, are degraded after they initiate tau polymerization [80]. There is strong support for both hypotheses from *in vitro* experiments as well as the *in vivo* studies discussed here.

To address whether amyloid deposition associated with AD perturbs the proteostasis network, APP^swe^/PS1dE9 transgenic mice with a high amyloid burden were investigated in order to determine whether cytosolic brain proteins would lose their solubility. Using a method that involved detergent extraction and sedimentation coupled with proteomic approaches, this study identified numerous cytosolic proteins that show specific losses in solubility as amyloid accumulates. The identified proteins included glycolytic enzymes as well as members of the 14-3-3 chaperone family. A substantial accumulation of lysine 48-linked polyubiquitin was also detected [81]. Furthermore, a recent study in *C. elegans* has shown that widespread protein aggregation is an inherent part of aging in worms [82], and by extension this could be assumed to hold true for humans.

The identification of cross-seeding raises the question of whether the neurodegeneration pathways are unique to different diseases or not. Transgenic *Drosophila* expressing human α-synuclein faithfully replicate essential features of human PD, including age-dependent loss of dopaminergic neurons, Lewy-body-like inclusions, and locomotor impairment. To define the transcriptional program involved in α-synuclein pathology, the expression of the entire *Drosophila* genome at pre-symptomatic, early, and advanced disease stages was determined. Fifty-one signature transcripts were tightly associated with α-synuclein expression, whereas in age-matched tau transgenic *Drosophila*, the transcription of α-synuclein-associated genes was normal, suggesting highly distinct pathways of neurodegeneration [83]. However, once the aggregates have formed, they have been shown to deregulate similar pathways and protein categories. This is the case even for proteins that aggregate in different organs. In support of this, Aβ and the amyloidogenic protein amylin, which forms aggregates in the pancreas of patients with type 2 diabetes, deregulate the same functional categories in cell culture systems; in fact, these two toxic molecules even show a great overlap in deregulated proteins [84].

Together, the findings from animal experimentation and the analysis of human brain tissue support *in vitro* studies revealing comorbidity in neurodegenerative disease. These studies highlight the involvement of both cross-seeding of the aggregating proteins, synergistic effects in their toxicity, and a vicious cycle of compromised aggregate clearance and protein aggregation.

## Note

This article is part of a series on *Cerebral multi-morbidity of the aging brain* edited by Johannes Attems and Julie Schneider. Other articles in the series can be found at http://alzres.com/series/cerebral_multimorbidity.

## Abbreviations

0N4R: Tau isoform with no amino-terminal insert and four microtubule-binding domains
AD: Alzheimer’s disease
ALS: Amyotrophic lateral sclerosis
APP: Amyloid precursor protein
Aβ: Amyloid-β
DLB: Dementia with Lewy bodies
FTDP-17t: Frontotemporal dementia linked to the tau gene on chromosome 17
FTLD: Frontotemporal lobar degeneration
FUS: Fused in sarcoma
GSK-3β: Glycogen synthase kinase 3β
MAP2/4: Microtubule-associated protein 2/4
MAPT: Microtubule-associated protein tau
NFT: Neurofibrillary tangle
PD: Parkinson’s disease
pff: pre-formed fibril
SAMP: Senescence-accelerated mice prone
SAMR: Senescence-accelerated mouse resistant
TDP-43: Transactive response DNA-binding protein 43 kDa
